# LPA rs10455872 polymorphism is associated with coronary lesions in Brazilian patients submitted to coronary angiography

**DOI:** 10.1186/1476-511X-13-74

**Published:** 2014-04-29

**Authors:** Paulo CJL Santos, Carolina T Bueno, Pedro A Lemos, José E Krieger, Alexandre C Pereira

**Affiliations:** 1 Laboratory of Genetics and Molecular Cardiology, Heart Institute (InCor), University of Sao Paulo Medical School, Av. Dr. Enéas de Carvalho Aguiar, 44 Cerqueira César, São Paulo, SP CEP 05403-000, Brazil; 2 Hemodynamic Laboratory, Heart Institute (InCor), University of Sao Paulo Medical School, Sao Paulo, Brazil

**Keywords:** *LPA* gene, rs10455872, rs3798220, Coronary lesions, Coronary artery disease

## Abstract

**Background:**

Polymorphisms in the *LPA* gene were associated with coronary artery disease (CAD). However, there are differences in the allelic frequencies, Lp(a) levels, and significant association with CAD according to ethnic groups. In this scenario, the main aim of this study was to assess the influence of the *LPA* polymorphisms on coronary lesions in Brazilian patients.

**Methods:**

1,394 consecutive patients submitted to coronary angiography to study suggestive CAD and twenty coronary segments were scored. Genotyping for the *LPA* rs10455872 and rs3798220 polymorphisms were performed by high resolution melting analysis.

**Results:**

The frequencies of the rs10455872 G and rs3798220 C variant alleles were 6.4% and 6.2%, respectively. *LPA* rs10455872 G variant allele was associated with higher odds ratio of having coronary lesions in an adjusted model (OR = 2.02, 95% CI = 1.10-3.72, p = 0.02). Scores of coronary lesions (extension, severity, and Gensini scores) were significantly different among rs10455872 genotype groups. Coronary lesions was not associated with *LPA* rs3798220 (OR = 1.09, 95% CI = 0.67-1.76, p = 0.73) and scores of coronary lesions were not different among rs3798220 genotypes.

**Conclusions:**

We confirmed the association of the *LPA* rs10455872 with CAD in a large sample of Brazilian patients. For the *LPA* rs3798220, our finding is consistent with studies which showed the lack of this genetic association.

## Introduction

Lipoprotein(a) [Lp(a)] is a plasma lipoprotein synthesized by the liver that is composed of a low-density lipoprotein (LDL) molecule, a high molecular weight glycoprotein apolipoprotein(a), and a single molecule of apolipoprotein(B). Physiological and pathogenic roles of Lp(a) remain partially unknown. Studies have suggested that Lp(a) provides a link between the cholesterol transport and the fibrinolytic system acting as a modulator of the blood clotting and fibrinolysis systems [1,2].

The increased concentration of Lp(a) has been associated with incidence and severity of cardiovascular disease (CVD), coronary artery disease (CAD), peripheral artery disease, and stroke [3-7]. A meta-analysis of published data from 31 prospective studies reported a relative risk for coronary heart disease of 1.60 (95% CI = 1.38-1.85) associated with Lp(a) levels [8,9].

More than 90% of the variance of Lp(a) concentration is explained by genetic variation [1]. A genome-wide association study showed that there is a group of genes strongly associated with CAD, such as solute carrier family 22 member 3 (*SLC22A3*), lipoprotein(a)-like 2 (*LPAL2*), and lipoprotein(a) (*LPA*), but investigators did not identify the functional variants at these loci [10-12].

Two polymorphisms in the *LPA* gene (rs3798220 and rs10455872) were associated with risk for CAD. However, there are differences in the allelic frequencies, Lp(a) levels, and degree of association with CAD according to ethnic groups [13-18]. In this scenario, the main aim of this study was to assess the influence of the *LPA* polymorphisms on coronary lesions in Brazilian patients submitted to coronary angiography.

## Patients and methods

### Patients submitted to coronary angiography

One thousand three hundred and ninety-four consecutive patients submitted to coronary angiography to study suggestive CAD were selected at the Laboratory of Hemodynamic, Heart Institute (InCor), Sao Paulo, Brazil. All patients had a clinical diagnosis of angina pectoris and stable angina. No patient enrolled in this study was currently experiencing an acute coronary syndrome. Patients with heart failure classes III–IV, hepatic dysfunction, familiar hypercholesterolemia, previous heart or kidney transplantation, and in antiviral treatment were excluded [19,20]. All patients signed an informed consent form and the protocol was approved by the ethics committee from Hospital das Clínicas from São Paulo University (CAPPesq 0398/04).

### Demographic data and laboratory tests

Data regarding general characteristic, weight, height, race/color, main cardiovascular risk factors (hypertension, diabetes, obesity, dyslipidemia, smoking, and current medical treatment) were obtained by interview. Race/color was classified as White, Brown (*Pardo* in Portuguese; person with admixture between White and Black), Black or Asiatic [21].

Triglycerides, total cholesterol (TC), high-density lipoprotein cholesterol, LDL cholesterol, and glucose were evaluated by standard techniques in 12-h fasting blood samples. Diabetes mellitus was diagnosed by the presence of fasting glucose ≥ 126 mg/dL or the use of antidiabetic drugs [22]. Hyperlipidemia was defined as TC ≥ 240 mg/dL, LDL-C ≥ 160 mg/dL, and/or use of lipid-lowering drugs [23].

### Hemodynamic and angiographic data

Blood pressure was measured in the sitting position with the use of a standard mercury sphygmomanometer on the left arm after 5 min rest. The first and fifth phases of Korotkoff sounds were used for systolic blood pressure (SBP) and diastolic blood pressure (DBP), respectively. The SBP and DBP were calculated from two readings with a minimal interval of 10 min apart. Hypertension was defined as mean SBP ≥140 mm Hg and/or DBP ≥90 mm Hg and/or antihypertensive drug use [24].

Twenty coronary segments were scored: each vessel was divided into three segments (proximal, medial, and distal), except for the secondary branches of the right coronary artery (posterior ventricular and posterior descending), which were divided into proximal and distal segments. Stenosis higher than 50% in any coronary segment was graded 1 point and the sum of points for all 20 segments constituted the Extension Score. Lesion severity was calculated as follows: none and irregularities, 0 points; <50%, 0.3 points; 50–70%, 0.6 points; >70–90%, 0.8 points; and >90–100%, 0.95 points. The Severity Score was calculated through the sum of points for all 20 coronary segments [25].

### Genotyping

Genomic DNA from subjects was extracted from peripheral blood following standard salting-out procedure. Additional file 1: Figure S1 shows genotyping detected by polymerase chain reaction (PCR) followed by high resolution melting (HRM) analysis with the Rotor Gene 6000® instrument (Qiagen, Courtaboeuf, France). The QIAgility® (Qiagen, Courtaboeuf, France), an automated instrument, was used according to instructions to optimize the sample preparation step [26].

Amplification of the fragment for the *LPA* rs10455872 (A > G, intron 25) polymorphism was performed using the primer sense 5′- ATGGGCTGGCAACACATAG -3′ and antisense 5′- CACTTTCTCCTCTAACCTGTATAA -3′ (78 pairs base). Amplification of the fragment for the *LPA* rs3798220 (T > C, p.Ile1891Met) polymorphism was performed using the primer sense 5′- GGCTCCAAGAACAGCCTAGA -3′ and antisense 5′- TCCTCAAGGCCTTCATCCTA -3′ (104 pairs base). A 40-cycle PCR was carried out with the following conditions: denaturation of the template DNA for first cycle of 94°C for 120 s, denaturation of 94°C for 20 s, annealing of 56.7°C for 20 s, and extension of 72°C for 22 s. PCR was performed with addition of fluorescent DNA-intercalating SYTO9® (1.5 μM; Invitrogen, Carlsbad, USA). In the HRM phase, the Rotor Gene 6000® measured the fluorescence in each 0.1°C temperature increase in the range of 72-81°C. Melting curves were generated by the decrease in fluorescence with the increase in the temperature; and in analysis, nucleotide changes result in three different curve patterns (Additional file 1: Figure S1). Samples of the three observed curves were analyzed using bidirectional sequencing as a validation procedure (ABI Terminator Sequencing Kit® and ABI 3500XL Sequencer® - Applied Biosystems, Foster City, CA, USA) [27]. The two methods gave identical results in all tests. The wild-type, heterozygous and mutant homozygous genotypes could be easily discernible by HRM analysis. In addition, 4% of the samples were randomly selected and reanalyzed as quality controls and gave identical results.

### Statistical analysis

Categorical variables are presented as percentage while continuous variables are presented as mean ± standard deviation. Chi-square test was performed for comparative analysis of general characteristics and coronary lesion frequency according to *LPA* polymorphisms. Dominant models (AG + GG) for the rs10455872 or (TC + CC) for the rs3798220 were performed because the frequencies of the GG and CC homozygous genotypes are low. Student’s t-test was performed for comparing the age, body mass index (BMI), biochemical data, blood pressures, and angiographic data means according to *LPA* polymorphisms. Biochemical data, blood pressures, and angiographic data were adjusted for age, gender, and race/color. Logistic regression univariate and multivariate analyses were performed to evaluate OR (odds ratio) for coronary lesions. Two adjusted models were performed: one using age, gender, and race/color and another with the additional covariates BMI, hyperlipidemia, statin use, and smoking. Linkage disequilibrium, Hardy-Weinberg equilibrium, and haplotype analyses were conducted with Haploview 4.0. All statistical analyses were carried out using the SPSS software (v. 16.0), with the level of significance set at p ≤ 0.05.

## Results

### General characteristics and LPA polymorphisms

Of the 1,394 patients, mean age of 59.7 ± 10.4, 1,126 (80.8%) had coronary lesions indicated by coronary angiography. The frequency of the rs10455872 G variant allele was 6.4% and the distribution of the genotypes was 0.6% (n = 8) for variant homozygous, 11.7% (n = 163) for heterozygous and 87.7% (n = 1223) for wild-type. The frequency of the rs3798220 C variant allele was 6.2% and the distribution of the genotypes was 0.4% (n = 6) for variant homozygous, 11.6% (n = 165) for heterozygous and 88.0% (n = 1248) for wild-type. The genotypic distribution for the *LPA* rs10455872 and rs3798220 polymorphisms were in accordance with the Hardy–Weinberg equilibrium (X^2^ = 1.01, p = 0.31 and X^2^ = 0.05, p = 0.82, respectively). Linkage disequilibrium analysis shows that the *LPA* rs3798220 and rs10455872 variant alleles had no strong disequilibrium (LD = 73) (Additional file 2: Figure S2).

### Biochemical, hemodynamic, and angiographic data according to LPA rs10455872 polymorphism

Table 1 shows data from patients submitted to coronary angiography according to *LPA* rs10455872 genotypes. The frequency of the *LPA* rs10455872 AG or GG genotypes was lower in non-Whites compared with Whites (p = 0.02). Patient carrying AG or GG genotypes had higher TC mean and proportion of hyperlipidemia. Regarding the angiographic data, higher frequency of coronary lesions (p = 0.004) was found in patients with AG or GG genotypes (88.9%) compared to patient with AA genotype (79.6%). Also, scores of coronary lesions (extension, severity, and Gensini scores) were significantly different among genotype groups (p < 0.001, p < 0.001, and p = 0.05, respectively) (Table 1).

**Table 1:** **General characteristics**, **biochemical**, **hemodynamic**, **and angiographic data according to *LPA *** **rs10455872 genotypes in the patients submitted to coronary angiography**

	**Genotypes**		***p*** **value**
**(n=1394,100%)**	**AA(n=1223)**	**AG+GG(n=171)**	
**Age(years)**	59.9 ± 10.1	59.5 ± 10.8	0.62
**Gender,female(%)**	40.6	37.4	0.42
**Race/color(%)**			
**White**	65.0	74.9	
**Intermediate**	29.8	23.4	0.02
**Black**	5.2	1.8	
**Hypertension(%)**	70.0	68.4	0.68
**Diabetes(%)**	31.2	27.5	0.33
**Hyperlipidemia(%)**	57.2	71.3	0.002
**Statin use(%)**	28.0	30.5	0.61
**Smokers(%)**	35.6	38.0	0.44
**Body mass index(Kg/m**^**2**^**)**	27.7 ± 4.8	27.6 ± 4.9	0.99
**Total cholesterol(mg/dL)**	228 ± 49	237 ± 46	0.05
**LDL-C(mg/dL)**	147 ± 44	155 ± 39	0.11
**HDL-C(mg/dL)**	42 ± 12	42 ± 10	0.82
**Triglycerides(mg/dL)**	183 ± 130	179 ± 108	0.75
**Systolic blood pressure(mmHg)**	149 ± 34	152 ± 39	0.61
**Diastolic blood pressure(mmHg)**	82 ± 15	84 ± 15	0.29
**Ejection fraction(%)**	60.6 ± 14.4	56.8 ± 17.7	0.07
**Coronary lesions(%)**	79.6	88.9	0.004
**Extension score**	2.1 ± 1.6	2.6 ± 1.7	<0.001
**Severity score**	1.5 ± 1.2	1.9 ± 1.3	<0.001
**Gensini score**	19.7 ± 28.2	25.7 ± 32.0	0.05

HDL-C: high density lipoprotein; LDL-C: low density lipoprotein.

Biochemical data, blood pressures, and angiographic scores were adjusted for age, gender, and race/color.

Coronary lesions frequency was compared between normal coronary arteries versus one-vessel, two-vessel, and three-vessel disease.

Furthermore, the presence of the *LPA* rs10455872 G variant allele was associated with higher OR of having coronary lesion, which we compared normal coronary arteries versus one-vessel, two-vessel, and three-vessel disease. Table 2 shows three models, being the OR of 2.02 (95% CI = 1.10-3.72, p = 0.02) in an adjusted model with age, gender, race/color, BMI, hyperlipidemia, statin use, and smoking. Also, Additional file 3: Table S1 shows logistic regression univariate analysis of the OR for coronary lesions. Additional file 4: Table S2 shows significant association for the variables age (as a continuous variable presented in the Table or as categorical variable - median age of 61 years as a cut-off – resulting in an OR of 1.88, 95% CI = 1.43-2.47, p < 0.001), gender, BMI, statin use, and hyperlipidemia in a logistic regression multivariate analysis.

**Table 2:** **Analysis of the coronary lesions odds ratio associated with *LPA *** **rs10455872 AG or GG genotypes in the patients submitted to coronary angiography**

**(n=1394,100%)**	**OR**	**95%CI**	***p*** **value**
**Models**			
**Unadjusted**	2.04	1.24-3.36	0.005
Adjusted*	2.05	1.22-3.43	0.006
Adjusted**	2.02	1.10-3.72	0.02

Coronary lesions frequency was compared between normal coronary arteries versus one-vessel, two-vessel, and three-vessel disease.

*Adjusted for age, gender, and race/color.

**Adjusted for age, gender, race/color, body mass index, hyperlipidemia, statin use, and smoking.

### Analysis stratified by race for the LPA rs10455872 polymorphism

In the White group (n = 901), patients with AG or GG genotypes (n = 128) had higher frequency of coronary lesions compared to patient with AA genotype (n = 773) (90.6% and 81.0%, respectively, p = 0.008). The presence of the *LPA* rs10455872 G variant allele was associated with higher OR of having coronary lesion in an adjusted model (OR = 2.17, 95% CI = 1.04-4.61, p = 0.03). Also, scores of coronary lesions were significantly different among genotype (extension: 2.7 ± 1.6 and 2.2 ± 1.6; severity: 1.9 ± 1.3 and 1.6 ± 1.2; and Gensini scores: 26.3 ± 30.0 and 19.7 ± 29.8) (p = 0.001, p = 0.01, and p = 0.04, respectively).

In the non-White group (n = 460, formed for Black and Brown individuals), frequency of coronary lesions was not statistically different (83.7% for AG or GG genotypes and 77.0% for AA genotype, p = 0.30). However, the presence of the G variant allele was associated with higher OR (OR = 1.95, 95% CI = 1.09-4.39, p = 0.04 – adjusted model) and scores of coronary lesions were different between AG or GG and AA genotypes (extension score: 2.6 ± 1.7 and 2.0 ± 1.6; and severity score: 1.9 ± 1.3 and 1.4 ± 1.2) (p = 0.04, and p = 0.03, respectively). Gensini score was marginally different between genotypes (24.7 ± 29.6 and 21.2 ± 27.5; p = 0.06).

### Biochemical, hemodynamic, and angiographic data according to LPA rs3798220 polymorphism

The proportion of hyperlipidemia was not different among rs3798220 genotypes (p = 0.62). Regarding the angiographic data, the frequency of coronary lesions was not associated with rs3798220 (p = 0.57) and no significant OR was observed in an adjusted model (OR = 1.09, 95% CI = 0.67-1.76, p = 0.73). Also, scores of coronary lesions (extension, severity, and Gensini scores) were not significantly different among genotype groups (p = 0.85, p = 0.56, and p = 0.46, respectively).

## Discussion

The two *LPA* polymorphisms studied in this study are some of the most important genetic markers for CAD. In this context, our main finding was that the *LPA* rs10455872 polymorphism is associated with coronary lesions in Brazilian patients submitted to coronory angiography. On the other hand, no association for the *LPA* rs3798220 was observed for any of the tested phenotypes.

Studies have reported higher Lp(a) concentration in sub-Saharan African descent and lower Lp(a) concentration in European descent [13,15,28,29]. Regarding the ethnicity, Brazil has one of the most heterogeneous population of the world, composed by a mixture of different ethinic groups, mainly European descent, African descent and Amerindians. In our data, a stratified analysis by race supported a role for the rs10455872 polymorphism independent of ethnic group.

Corroboring with our study, Anderson et al. found that the rs10455872 polymorphism strongly predicted prevalent CAD (per allele OR = 1.43, 95% CI = 1.07-1.91) [30]. Helgadottir et al. showed that patients with CAD carrying *LPA* risk alleles have increased susceptibility to atherosclerotic manifestations outside of the coronary tree and they are more likely to be diagnosed earlier with CAD than are CAD cases not carrying this variant [31]. Other studies that analyzed the rs10455872 and rs3798220 polymorphisms together reported an increased risk of coronary disease and Lp(a) level that can be explained by these *LPA* polymorphisms [11,32]. *LPA* rs10455872 is an intronic polymorphism associated with short KIV-2 repeat region (kringle IV type 2) which is associated with Lp(a) levels. In the present study, the frequency of the rs10455872 G variant allele was 6.4% for overall, but we observed higher allelic frequency in White compared with non-White groups. The association of the rs10455872 with CAD was significant even in the non-White group which had a small sample size. These data suggest that rs10455872 is also a strong genetic marker for CAD risk in ethnically mixed populations.

For the *LPA* rs3798220 polymorphism, we did not observe significant association with coronary lesions. Data from other studies also did not support a relationship between this *LPA* variant and CAD [33,34]. Furthermore, Anderson et al., studying 1,400 participants with coronary angiography (more than 90% Whites), did not find an association signal between rs3798220 and CAD (OR = 1.47, 95% CI = 0.81-2.67, p = 0.20) [30]. In contrast to our study, the rs3798220 has previously been reported to have an association with the Lp(a) level and the risk of coronary disease [35-37]. *LPA* rs3798220 results in an aminoacid substitution in the protease domain of *LPA*, but it can not provide stronger association than rs10455872 which might be representating a more complex group of genetic variants or repeat structures. A possible hypothesis for the lack of this association in the present study could be the lower value of linkage disequilibrium between rs3798220 and rs10455872 identified in the Brazilian patients compared with some studies with patients predominantly from European descent [11,35-37]. Another hypothesis may be low statistical power, but less likely if the impact of rs3798220 was approximately equal to the impact of rs10455872. However, the exact reason is unclear and other genetic components differently expressed due to ethnicity might be important modulators.

The exact mechanism by which an increased Lp(a) level increases the CAD risk is not fully understood. Pathways modulated by Lp(a) may involve the LDL-cholesterol transport system, the inhibition of the expression of tissue factor, the inhibition of conversion of plasminogen to plasmin, the carriage of pro-inflammatory oxidized phospholipids, and an atherosclerotic stenotic mechanism [30,38-44]. Some studies reported that the severity of coronary artery disease is associated with Lp(a) levels or LDL concentration [45-48]. Regarding to the Lp(a) level as a risk factor in different ethnic groups, Lp(a) has been associated with risk in European populations [1], but not unequivocally in African Americans [17,18]. However, a recent study identified that the increased risk of CVD was at least as strong in African Americans as in White Americans [49]. Another study investigated differential frequencies of *LPA* polymorphisms in non-Hispanic whites, non-Hispanic blacks, and Mexican Americans [50]. Interestingly, 15 of the 19 polymorphisms tested were strongly associated with Lp(a) levels in at least one subpopulation, six in at least two subpopulations, and none in all three subpopulations. The lack of generalization of associations across ethnicities suggests that specific *LPA* variants may be contributing to the observed Lp(a) between-population variance. Authors also compared the allele frequencies in HapMap, and observed extremely high correlations (r ≥ 0.99) in allele frequencies between non-Hispanic whites and HapMap CEU (US individuals of northern and western European ancestry) and between non-Hispanic blacks and both HapMap YRI (Yoruba from West Africa) and ASW (individuals with African ancestry from the Southwest USA) [50].

There are some limitations in our study. First, we did not measure Lp(a) levels and we also did not genotype KIV-2 repeats to check their association with both the *LPA* polymorphisms and/or the CAD phenotype. Second, we did not assess ancestry through genetic markers; instead, we used a self-declared classification which is commonly applied in Brazil and correlates with genetic ancestry determination. In addition, in our stratified analysis by race, we observed significant association of the rs10455872 with CAD in the White and non-White patient groups. Third, our plaque burden data are derived from institutional records and represent real-life data, as opposed to core-lab derived hemodinamic data. Thus, and despite the greater external validity of our results, we were not able to determine inter- or intra-observed variability estimates. In addition, our choosen method for establishing atherosclerotic burden in the studied patients has relied upon the Gensini Score, which has been shown to highly correlate with this end-point. Other scores could also be used, although they are not as well fitted for quantifing plaque burden. One example is the Syntax score, an angiographic tool for grading the complexity of CAD and designed to better anticipate the risks of percutaneous or surgical revascularization. Finnaly, it is not possible to completely exclude the interaction of the covariates as other genetic markers, use of concomitant drugs, ethnicity, gender and age on our findings [51-54]. Nonetheless, our findings remained after multivariate analysis.

## Conclusions

In conclusion, we confirmed the association of the *LPA* rs10455872 with CAD in a large sample of Brazilian patients. For the *LPA* rs3798220, our finding is consistent with studies which showed the lack of this genetic association.

## Competing interests

The authors declare that they have no competing interests.

## Authors' contributions

PCJLS and CTB carried out molecular genetic analyses, performed the statistical analysis and drafted the manuscript. PCJLS, ACP, PAL, JEK conceived the study, and participated in its design. All authors read and approved the final manuscript.

## Supplementary Material

Additional file 1: Figure S1 Graphs of the *LPA* rs10455872 (A > G, intron 25) genotyping. Nucleotide changes results in different curve patterns using high resolution melting analysis. A: Graph of normalized fluorescence by temperature. B: Graph of normalized fluorescence (based on genotype 2) by temperature. 1: wild-type genotype (AA); 2: heterozygous genotype (AG); 3: mutant homozygous genotype (GG). Click here for file

Additional file 2: Figure S2 Linkage disequilibrium and haplotype analyses for the *LPA* rs3798220 and rs10455872 polymorphisms in the patients submitted to coronary angiography. Click here for file

Additional file 3: Table S1 Logistic regression univariate analysis of the coronary lesions odds ratio in the patients submitted to coronary angiography. Click here for file

Additional file 4: Table S2 Logistic regression multivariate analysis of the coronary lesions odds ratio in the patients submitted to coronary angiography. Click here for file
