# Supplementary material for: LPA rs10455872 polymorphism is associated with coronary lesions in Brazilian patients submitted to coronary angiography
Source: Lipids Health Dis. 2014 Apr 29;13:74. doi: 10.1186/1476-511X-13-74 (PMC4108154; doi:10.1186/1476-511X-13-74)
Supplement: Additional file 1: Figure S1 — Graphs of the LPA rs10455872 (A > G, intron 25) genotyping. Nucleotide changes results in different curve patterns using high resolution melting analysis. A: Graph of normalized fluorescence by temperature. B: Graph of normalized fluorescence (based on genotype 2) by temperature. 1: wild-type genotype (AA); 2: heterozygous genotype (AG); 3: mutant homozygous genotype (GG). [file 1476-511X-13-74-S1.doc]

**Additional file 1: Figure S1. Graphs of the *LPA* rs10455872 (A>G, intron 25) genotyping.** Nucleotide changes results in different curve patterns using high resolution melting analysis. A: Graph of normalized fluorescence by temperature. B: Graph of normalized fluorescence (based on genotype 2) by temperature. 1: wild-type genotype (AA); 2: heterozygous genotype (AG); 3: mutant homozygous genotype (GG).
