# Supplementary material for: LPA rs10455872 polymorphism is associated with coronary lesions in Brazilian patients submitted to coronary angiography
Source: Lipids Health Dis. 2014 Apr 29;13:74. doi: 10.1186/1476-511X-13-74 (PMC4108154; doi:10.1186/1476-511X-13-74)
Supplement: Additional file 2: Figure S2 — Linkage disequilibrium and haplotype analyses for the LPA rs3798220 and rs10455872 polymorphisms in the patients submitted to coronary angiography. [file 1476-511X-13-74-S2.doc]

**Additional file 2: Figure S2. Linkage disequilibrium and haplotype analyses for the *LPA* rs3798220 and rs10455872 polymorphisms in the patients submitted to coronary angiography.**
