# Supplementary material for: LPA rs10455872 polymorphism is associated with coronary lesions in Brazilian patients submitted to coronary angiography
Source: Lipids Health Dis. 2014 Apr 29;13:74. doi: 10.1186/1476-511X-13-74 (PMC4108154; doi:10.1186/1476-511X-13-74)
Supplement: Additional file 3: Table S1 — Logistic regression univariate analysis of the coronary lesions odds ratio in the patients submitted to coronary angiography. [file 1476-511X-13-74-S3.doc]

**Additional file 3: Table S1. Logistic regression univariate analysis of the coronary lesions odds ratio in the patients submitted to coronary angiography**

**
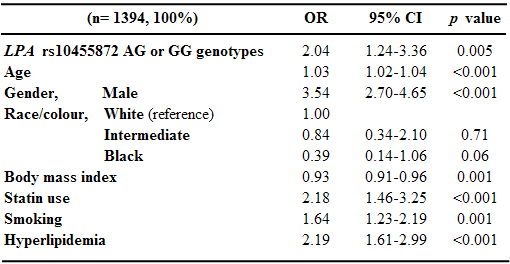
**
