# Supplementary material for: LPA rs10455872 polymorphism is associated with coronary lesions in Brazilian patients submitted to coronary angiography
Source: Lipids Health Dis. 2014 Apr 29;13:74. doi: 10.1186/1476-511X-13-74 (PMC4108154; doi:10.1186/1476-511X-13-74)

**Additional file 4: Table S2. Logistic regression multivariate analysis of the coronary lesions odds ratio in the patients submitted to coronary angiography**


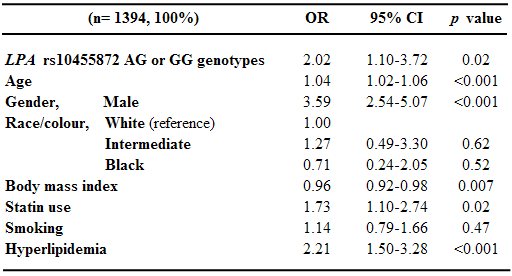

Supplement: Additional file 4: Table S2 — Logistic regression multivariate analysis of the coronary lesions odds ratio in the patients submitted to coronary angiography. [file 1476-511X-13-74-S4.doc]
